# Clinical and Radiological Features of an Adenovirus Type 7 Outbreak in Split-Dalmatia County, Croatia, 2022–2023

**DOI:** 10.3390/pathogens13121114

**Published:** 2024-12-17

**Authors:** Antea Trogrlic, Dina Mrcela, Danijela Budimir Mrsic, Ivana Jukic, Sanda Sardelic, Irena Tabain, Željka Hruskar, Diana Nonkovic, Josko Markic, Mirela Pavicic Ivelja

**Affiliations:** 1 Department of Infectious Diseases, University Hospital of Split, Spinciceva 1, 21000 Split, Croatia; trogrlicantea27@gmail.com; 2 Department of Pediatrics, University Hospital of Split, Spinciceva 1, 21000 Split, Croatia; mrceladina@gmail.com (D.M.); ijukic@kbsplit.hr (I.J.); 3 Department of Diagnostic and Interventional Radiology, University Hospital of Split, Spinciceva 1, 21000 Split, Croatia; danijelabudimir@gmail.com; 4 School of Medicine, University of Split, Soltanska 2a, 21000 Split, Croatia; 5 Department of Microbiology and Parasitology, University Hospital of Split, Spinciceva 1, 21000 Split, Croatia; ssardeli@kbsplit.hr; 6 Croatian Institute of Public Health, Rockefellerova 7, 10000 Zagreb, Croatia; irena.tabain@hzjz.hr (I.T.); zeljka.hruskar@hzjz.hr (Ž.H.); 7 Teaching Institute for Public Health of Split-Dalmatia County, Vukovarska 46, 21000 Split, Croatia; diana.nonkovic@nzjz-split.hr; 8 Department of Health Studies, University of Split, R. Boskovica 35, 21000 Split, Croatia

**Keywords:** adenovirus, Brixia score, pneumonia, pulmonary embolism

## Abstract

Human adenoviruses (HAdVs) are known to be highly contagious pathogens. They are commonly associated with mild respiratory infections in young children but can also cause severe life-threatening infections. Human adenovirus types 4 and 7 have frequently been reported to cause pneumonia in immunocompetent youths and adults. In this retrospective study, we analyzed the clinical, laboratory, radiological, and microbiological features, as well as the treatment and outcomes of an adenovirus outbreak in 185 patients who were admitted to the Emergency Unit of the Departments of Infectious Diseases and Pediatrics, University Hospital of Split, Croatia, between October 2022 and April 2023. An unusual increase in the frequency of adenovirus pneumonia was observed, especially in adults, followed by respiratory failure and complications such as pulmonary embolism. The most common chest X-ray findings were unilateral patchy opacity and unilateral reticulations (11.6%), followed by unilateral lobar pneumonia (7.1%). The predominant CT presentation was unilateral lobar pneumonia with multiple patchy ground glass opacities (23.5%) or lobar pneumonia with mixed opacities (17.6%). We found a low correlation between Brixia score and C-reactive protein in adults and no correlation in children. Adenovirus type 7 was almost exclusively isolated from patients with pneumonia. Most of our patients with severe or critical adenovirus pneumonia were immunocompetent adults without any medical history. So far, only a few studies have presented the radiological features of HAdV pneumonia, which generally did not reveal lobar pneumonia in a substantial percentage. Our research also demonstrated an unusual presentation of adenovirus infection complicated with pulmonary embolism, which has rarely been reported in previous studies. The aforementioned HAdV outbreak indicates the necessity for further research, especially in the context of effective antiviral therapy and infection prevention.

## 1. Introduction

Adenoviruses are DNA viruses commonly found in humans and animals. There are more than 100 serologically different types of adenoviruses, and some of them can cause disease in humans (HAdV) [1]. Adenoviruses can cause a range of clinical syndromes, from localized disease of the respiratory, gastrointestinal, and/or urinary tract to disseminated disease in both adults and children [2]. Transmission of HAdV can occur via aerosol droplets (sneezing and coughing), the fecal–oral route, ingestion of contaminated food or water, and by contact with contaminated fomites [3]. Adenoviruses can survive over seven days on environmental surfaces and medical instruments. Since they are non-enveloped, they are resistant to lipid disinfectants but are inactivated by heat, formaldehyde, or bleach [3,4]. Adenovirus can spread through water, for example, in swimming pools [5]. Transmissions during birth via exposure to cervical canal secretions and in solid organ transplants are very rare [6,7]. The airborne pathway likely contributes to the spread of adenovirus in closed or crowded settings, causing outbreaks of adenovirus infection. Outbreaks are common in daycare centers, schools, military camps, and prisons [8,9]. The wide spectrum of clinical diseases caused by HAdV is related to age, immune status, and population characteristics. Due to the lack of humoral immunity, adenovirus infection is more common in young children [10]. HAdV-associated disease is much more severe and can even be lethal in immunocompromised patients [11].

HAdVs are a family of >60 serotypes divided into seven subgroups or species (A through G). Of these, adenovirus serotypes 4 (HAdV-4) and 7 (HAdV-7) are often associated with upper and lower respiratory disease and conjunctivitis, which can be a clinical clue to the etiology of infection [12]. HAdV-7 is frequently associated with severe complications of adenoviral pneumonia [13]. Patients most often present with fever, myalgia, diarrhea, and vomiting with a worsening cough and dyspnea unresponsive to empiric antibiotic therapy [3]. The benefits of antiviral drugs such as acyclovir, ganciclovir, ribavirin, and cidofovir have been studied for usage in immunocompetent patients but have not been fully investigated [14,15].

A notable increase in the circulation of human adenoviruses (HAdVs) was observed in 2023 [16]. The reason for this may lie in the various non-pharmaceutical interventions implemented during the COVID-19 pandemic, which significantly affected the prevalence of common respiratory viruses and contributed to more complex patterns of respiratory virus circulation in the post-pandemic period [17]. During the end of 2022 and the beginning of 2023, an unusual number of severe cases of adenovirus infections were registered in Split-Dalmatia County in Croatia. The fact that it was unprecedented in this specific region suggests a possible combination of factors and the importance of understanding genetic interactions like recombination and mutations alongside ecological and biological factors. It is also important to consider that the zoonotic potential of certain viruses might be incomplete, leaving us vulnerable to unexpected outbreaks [18]. The increased frequency of pulmonary embolism in young adult male patients, a condition not previously described in the literature, was also noticed. Here, we present the clinical, laboratory, radiological, and microbiological features, as well as the treatment and outcomes of this adenovirus outbreak.

## 2. Materials and Methods

### 2.1. Study Design and Participants

We performed a retrospective, single-center study in children and adults with adenovirus infection who were admitted to the Emergency Unit of the Departments of Infectious Diseases and Pediatrics at the University Hospital of Split between 1 October 2022 and 30 April 2023. All patients were from Split-Dalmatia County in Croatia. Ethical approval was granted by the Ethics Committee of the University Hospital of Split (500-03/23-01/90) on 1 April 2023. The analyzed parameters were the following: the number of adenovirus cases per month, the demographic and clinical characteristics of infected patients (gender, age, weight, smoking, comorbidities, date of symptom onset, clinical manifestations, complications, hospitalization rate, outcome, therapy, and length of hospital stay). The laboratory parameters analysis encompasses hematological parameters (including total leukocyte, neutrophil, thrombocyte, and lymphocyte counts), C-reactive protein (CRP) levels, liver and renal functional tests, lactate dehydrogenase, creatinine kinase, sodium levels, and D-dimer. Imaging modalities of the lung (chest X-ray-CXR and computer tomography-CT) were also analyzed, as well as the individual characteristics of patients with pulmonary embolism.

### 2.2. Definitions

Patients with adenovirus pneumonia were divided by the clinical presentation severity into three groups: mild, severe, and critical, based on the following criteria. Mild pneumonia was pneumonia without the need for oxygen therapy. Severe pneumonia was defined as pneumonia that required oxygen therapy (with hypoxemia, SpO_2_ < 90%), while critical was pneumonia that required mechanical ventilation (MV) or admission to the intensive care unit (ICU) [19,20].

In all patients where the CXR was performed, we calculated the Brixia score, an X-ray severity scoring system designed for COVID-19 pneumonia, which shows the extent of lung changes in six lung regions [21,22]. Each region is scored from 0 to 3 [(0—no abnormalities, 1—interstitial infiltrates, 2—interstitial and alveolar infiltrates (interstitial predominance), and 3—interstitial and alveolar infiltrates (alveolar predominance)], with a total score from 0 to 18. The sum of scores across all six regions indicates the overall severity of lung involvement. Based on the previous researches, we classified our patients into 4 groups according to the value of the Brixia score: normal (score 0), mild (score from 1 to 6), moderate (score from 7 to 12), and severe (score from 13 to 18) [21,23]. Serial scoring can be used to track changes in lung involvement over time and adjust treatment plans as necessary.

In patients where a CT chest was requested, we calculated the CT chest involvement score, which is the sum of the individual lobar scores [24]. It is classified as follows: Score-1 (<5% area involved), Score-2 (5–25% area involved), Score-3 (25–50% area involved), Score-4 (50–75% area involved), and Score-5 (>75% area involved), making the total score 25 (maximum involvement). The severity of the lung involvement on the CT correlates with the severity of the disease. According to this criterion, we created three groups of patients depending on the total value of the CT involvement score: score <8 (mild), score from 9 to 15 (moderate), and score >16 (severe) [25].

The Charlson Comorbidity Index (CCI) was calculated for all patients according to the scoring tables published by Charlson et al. [26]. It is used as a method of assessing the risk of death from comorbid diseases. Based on the severity of comorbid diseases, we categorized the CCI scores into three groups: mild, with CCI scores of 1–2; moderate, with CCI scores of 3–4; and severe, with CCI scores ≥5. Higher scores indicate a greater burden of comorbid conditions and, consequently, a higher risk of adverse health outcomes, including death.

The outcome is divided into three groups. The first favorable group includes patients who did not need oxygen therapy or only required low levels of oxygen flow. Those patients had a mild to moderate course of illness with stable respiratory function and recovered without significant medical intervention. The second group includes patients with less favorable outcomes. Patients in this category were on mechanical ventilation, suggesting a more severe condition where the patient’s respiratory function was compromised and advanced support was necessary to maintain life. This group reflects a more serious illness that required intensive care. The third fatale group refers to patients who died as a result of their condition.

### 2.3. Testing for Adenovirus

We screened patients who presented with fever, respiratory symptoms, gastrointestinal symptoms, or conjunctivitis, or had epidemiological information about contact with patients who had confirmed adenovirus infection using a rapid antigen test for adenovirus infection. We tested with Rapid Adenovirus & RSV Antigen Nasal Test Kit, Ecotest, Hague, The Netherlands. All hospitalized patients had their diagnosis additionally confirmed by polymerase chain reaction (PCR). Also, patients who were negative on the rapid antigen test and highly suspected of adenovirus infection underwent a respiratory multiplex polymerase chain reaction test. Using multiplex PCR, we enhanced the diagnostic accuracy by broadening the scope of detection. For both tests, we collected nasopharyngeal swabs and/or aspirates on the day of admission or the following day if the patient was hospitalized.

#### 2.3.1. Molecular Detection of Adenoviruses

DNA was extracted from a 200 µL sample using automated DNA/RNA extraction (GeneRotex 96 Nucleic Acid Extractor, Tianlong Science and Technology, Xi’an, China). The Multiplex–Tandem (MT) PCR test for identifying respiratory viruses was conducted with the commercial panel of assays Respiratory viruses (16-well) that included 12 pathogens: Enteroviruses, Parechovirus, Rhinoviruses, Human Bocavirus, Influenza A and B, Respiratory Syncytial Virus, Human Parainfluenza 1–4, Human Metapneumovirus, Human Adenovirus, Human Coronavirus, and SARS-CoV-2 (AusDiagnostics, Mascot, Sydney, Australia; BioFire Respiratory Panel 2.1 plus (RP2.1 plus, BioFire Diagnostics LLC (bioMerieux) Salt Lake City, UT, USA). Due to the fact that the adenovirus detection was done by a commercial kit (Quanty Adenovirus (hexon gene), CLONIT, Milano, Italy), no primer sequence is provided in the user manual.

#### 2.3.2. Virus Isolation in Cell Culture

HeLa cell line was used for the viral propagation of PCR-confirmed samples. The cell culture was maintained using a Minimum essential medium (MEM) supplemented with 2% fetal bovine serum (Capricorn Scientific GmbH, Ebsdorfergrund, Germany), L-glutamine (2 mM), penicillin (200 U/mL), and streptomycin (200 μg/mL). The sample (0.2 mL) was inoculated into duplicate cell culture tubes and incubated at 37 °C for 1–7 days. The cells were observed for cytopathic effect (CPE) daily for 7 days. The virus isolates were kept frozen at −80 °C.

#### 2.3.3. Microneutralization Test

The virus isolates were further typed with a microneutralization test using type-specific antisera that can detect 8 serotypes (HAdV 1–7 and 14). Virus titer (TCID 50) was calculated using the Spearman–Kärber method. [21] 50 µL of virus isolates (concentration 100 TCID 50/50 µL) were added to 96-well plates and mixed with 50 µL type-specific HAdV antibodies. The mixture was incubated at 37 °C for 1 h. After incubation, 100 µL of HeLa cell suspension (2.5 × 10.5 cells/mL) was added to each well, and the plates were then incubated at 37 °C in a 5% CO_2_ for up to 7 days. The presence of CPE was observed daily. The specific serotype was detected by 70–100% inhibition of the cytopathic effect in wells containing type-specific antibodies.

### 2.4. Statistical Analysis

Statistical data analyses were performed using JASP (Jeffrey’s Amazing Statistics Program) version 0.16.2 (JASP Team, Amsterdam, The Netherlands), IBM SPSS software (version 28.), and Python (version 3.9.13). The normality of the distribution of numerical variables was tested using the Shapiro–Wilk test. Median (Md) and interquartile range (IQR) were used for quantitative non-normally distributed variables. To describe the distribution of categorical variables in our study, relative and absolute frequencies were used. Comparative analyses were performed using the Chi-square test (χ^2^) for the categorical variables and the Mann–Whitney U-test for the numerical variables. When, in a certain cell, the frequency of the event was low, Fisher’s exact test was used instead. We evaluated the correlation between the Brixia score and laboratory parameters using Spearman’s correlation coefficient. All analyses with *p* values < 0.05 or at a 95% confidence level were considered statistically significant.

## 3. Results

**Figure 1:**
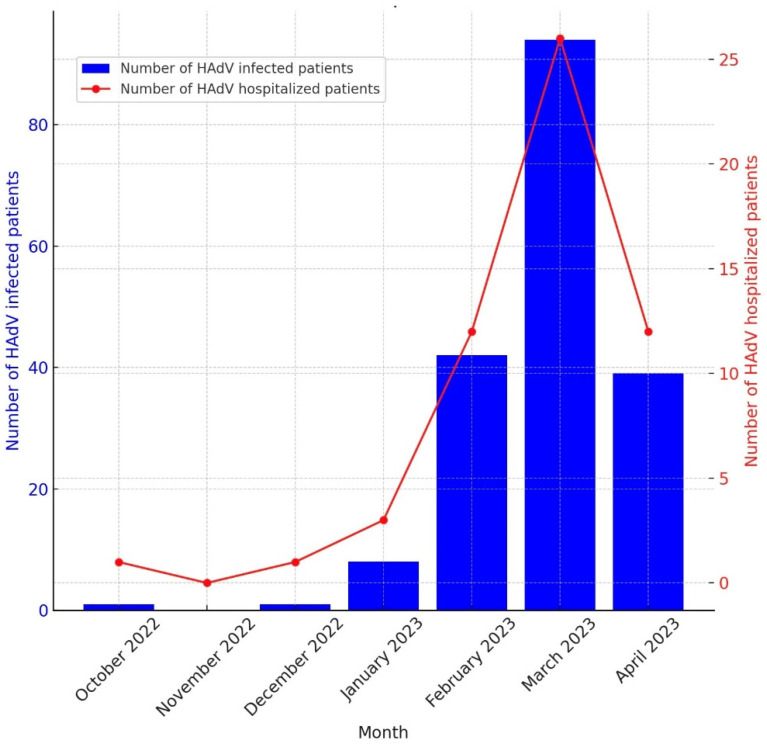
Month-by-month distribution of human-adenovirus-positive infections. Blue bar: number of HAdV-infected patients. Red line: number of HAdV-hospitalized patients.

The study included 185 patients with confirmed HAdV infection: 52 adults (28.1%) and 133 (71.9%) children, with ages ranging from 1 month to 59 years. Among these cases, 109 (58.9%) were diagnosed in the Department of Infectious Diseases and 76 (41.1%) in the Department of Pediatrics of the University Hospital of Split. Hospitalization was required for 55 patients (29.7%), including 29 adults (52.7%) and 26 children (47.3%). The monthly distribution of the detected adenovirus cases between October 2022 and April 2023 and the number of hospitalized cases are shown in Figure 1. In March 2023, a significant increase in both the HAdV-positive cases and HAdV-related hospitalizations was observed, in contrast to the absence of recorded HAdV infections in November 2022.

The patient demographic, clinical, and laboratory characteristics are shown in Table 1.

Regarding the comorbidities, 2 adults had hypertension, 1 had diabetes, and a total of 4 patients (2 adults and 2 children) had chronic lung disease. Obesity was noted in 7 patients, including 1 child. Among the cohort, 23 adults and 2 older minors were identified as smokers. The most common clinical manifestation of HAdV infection in children was acute tonsillitis, with 80 (60.2%) cases, while in adults, it was confirmed in 19 (36.5%) patients. Compared to children, where pneumonia was diagnosed in 14 (10.5%) cases, in adults, it was detected in 35 (67.3%) patients. In addition to that, the higher incidence of pneumonia resulted in a higher adult hospitalization rate compared to children [29 (55.8%) vs. 26 (19.5%), *p* < 0.001]. However, there was no statistically significant difference in length of hospital stay (*p* = 0.194) between adults and children (Table 1).

The virus isolation was carried out on 26 samples from pneumonia patients (14.05% of the cohort). Among these, 50% (13/26) were identified as HAdV-7, one sample (3.8%) as HAdV-3, while 12 samples (46.2%) were inconclusive in the neutralization test. Sequencing was successful for one HAdV7 isolate.

The laboratory results are shown in Table 2, revealing significant differences between adults and children regarding almost all the parameters observed.

Chest X-ray (CXR) was performed on 112 patients, including 46 adults (41.1%) and 66 children (58.9%) (Table 3). Of these, 57 CXRs (50.9%) were reported as normal, while 55 (49.1%) revealed pathological findings, with abnormal CXRs significantly more frequent in adult patients (*p* < 0.001). The most common CXR findings were unilateral patchy opacity and unilateral reticulations (N = 13, 11.6%), followed by unilateral lobar pneumonia (N = 8, 7.1%). Additionally, a statistically significant difference was found in the distribution of Brixia score categories between adults and children (*p* = 0.018). Adults demonstrated a significantly higher median Brixia score than children (*p* < 0.001), indicating a greater severity of lung involvement. All CXR findings and the Brixia score index are summarized in Table 3.

**Figure 2:**
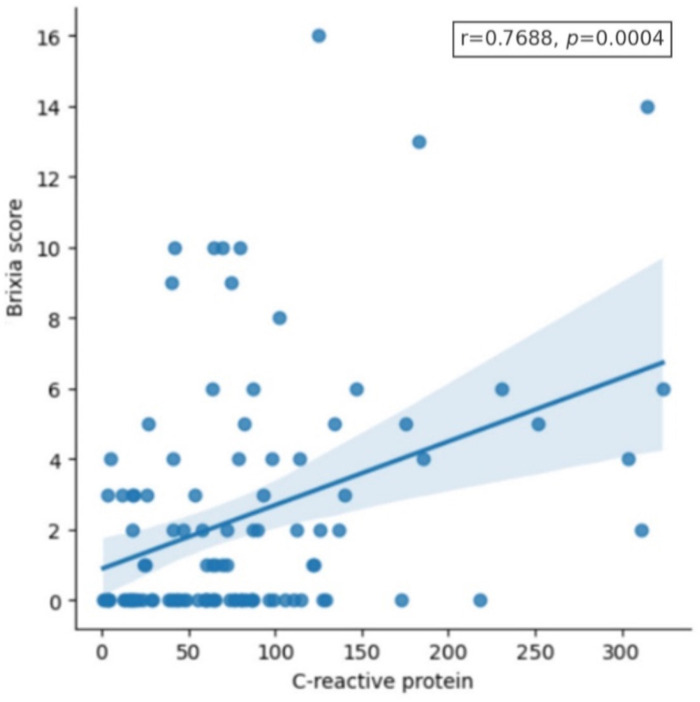
Correlation between Brixia score and C-reactive protein (mg/L) in patients’ samples.

**Figure 3:**
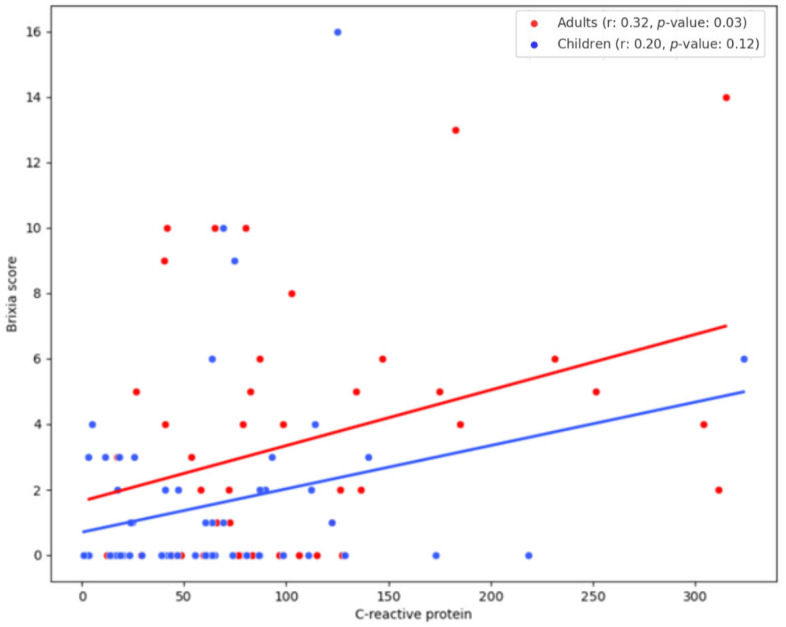
Correlation between Brixia score and C-reactive protein (mg/L) in children and adults.

The correlation between the Brixia score and CRP levels in the overall patient population, as well as separately for adults and children, is graphically shown in Figure 2 and Figure 3. A low correlation was observed between CRP and the Brixia score for all patients (*r* = 0.339; *p* < 0.001) and adults (*r* = 0.32; *p* = 0.03). In contrast to that, no significant correlation was found in children (*r* = 0.20; *p* = 0.12). Interestingly, several patients demonstrated elevated Brixia scores even though their CRP levels remained below 150 mg/L. The values of Spearman’s correlation coefficient and *p*-value for other laboratory parameters are summarized in Table 4. The correlation between some other laboratory parameters with the Brixia score was found to be weak/moderate, as shown in Table 4.

Chest computer tomography (CT) was performed on 17 patients, including 12 (70.6%) adults and 5 (29.4%) children. The findings from the chest CT scan, as well as the CT involvement score, are shown in Table S1. The findings from the chest CT scans, along with the CT involvement scores, are presented in Table S1. No statistically significant difference in findings and scores was observed between adults and children (*p* > 0.05).

The demographic and clinical characteristics of non-hospitalized and hospitalized HAdV-positive patients are summarized in Table S2. Non-hospitalized patients were generally younger and less frequently smokers or obese, with acute tonsillitis as the predominant diagnosis observed in 61.5% of cases. Pneumonia was identified in only 13 non-hospitalized patients (10%), all of whom experienced a mild clinical course, in contrast to 41 hospitalized patients (74.5%) who presented with more severe cases. The laboratory findings for both non-hospitalized and hospitalized HAdV-positive patients are summarized in Table S3, indicating that the hospitalized patients more frequently exhibited lower platelet counts, elevated levels of CRP, lactate dehydrogenase, creatine kinase, and D-dimer, along with markers of impaired liver function.

Table 5 shows the median (IQR) of the Brixia score index and the Brixia score category of hospitalized and non-hospitalized HAdV-positive patients. Notably, the median of the Brixia score index was significantly higher in hospitalized patients compared to non-hospitalized patients [3 (1.25, 6) vs. 0 (0, 1), *p* < 0.001].

Logistic regression was conducted to assess the effects of male sex, age, CRP, and Brixia scores on the HAdV infection outcome. The logistic regression model was statistically significant, χ^2^ (101) = 18.065, *p* < 0.001. Increasing Brixia score, CRP, as well as male sex, are associated with less favorable outcomes or death (Table 6).

Table 7 presents the differences in hospital length of stay among patients treated and not treated with antibiotics, ribavirin, and corticosteroids. A statistically significant difference was observed in the length of hospital stay between patients treated with corticosteroids and those not treated, with shorter stays noted in patients who did not receive corticosteroids (*p* = 0.012).

Table S4 presents the demographic, clinical, and laboratory individual characteristics of six male patients with adenovirus infection who developed pulmonary embolism during the period of hospitalization. In Table S5, we summarized their CXR and CT findings as well as the Brixia and CT involvement scores.

## 4. Discussion

In this study, we analyzed the clinical and demographic characteristics of adenovirus (HAdV) infections in both pediatric and adult patients, highlighting the differences observed in infection severity, radiologic findings, and clinical outcomes. Adenovirus infections are typically more common in children, a finding consistent with our study, which showed a high proportion of pediatric cases (71.9%) with male predominance (69.2%). This prevalence in younger patients is largely attributed to the lack of humoral immunity with lower levels of circulating neutralizing antibodies, as opposed to adults, who often have immunity from previous exposures [27,28]. Similar to the findings of this study, the previous research has demonstrated a higher prevalence of adenovirus infections among male patients, a pattern also observed with other respiratory viral infections, such as influenza, parainfluenza, and respiratory syncytial virus [27,29].

The clinical severity of HAdV infection during the outbreak in Split-Dalmatia County varied significantly among age groups, with a higher incidence of severe pneumonia and need for respiratory support observed among adults compared to children. Among the total of 185 patients, 55 (29.7%) required hospitalization, including 26 adults (47.3%) and 29 children (52.7%). Pneumonia was diagnosed in 35 adults and 19 children. Mechanical ventilation was required in two adult patients and four children. A 10-year-old child died. This indicates that HAdV pneumonia may present with life-threatening respiratory failure requiring support either through mechanical ventilation or extracorporeal life support [30]. In all but one collected swab with successful virus isolation and typing, HAdV type 7 was identified, which leads us to the conclusion that HAdV-7 was the cause of the adenovirus outbreak, with a significant number of severe pneumonia cases among children and young adults in our region. In the literature, adenovirus outbreaks associated with severe and critical pneumonia have been associated mostly with serotypes 3, 7, 14, and 55. In the previous study conducted on hospitalized HAdV-positive children from Zagreb, Croatia, the most common serotype detected was HAdV-2 [31]. In an outbreak in Nanjing, China, in 2016, 52 patients were found to have HAdV-7 pneumonia. In contrast to the Croatian outbreak, none of the patients in the previous study required mechanical ventilation, and only two patients received low-flow oxygen therapy [13]. This differs significantly from the Split-Dalmatia outbreak, where 15 out of 54 hospitalized patients with pneumonia necessitated oxygen support, and six patients presented with critical pneumonia requiring mechanical ventilation or admission to the intensive care unit (ICU). In 2014, an adenovirus outbreak occurred in Oregon, USA, where HAdV-B7 was the most commonly detected serotype, identified in 59% of cases. Among the 198 patients, 80 (40.4%) were adults and 118 (59.6%) were children. A total of 158 patients (79.8%) were hospitalized, with mechanical ventilation required for 25 patients, and five patients died [32]. In the current study, the majority of patients were immunocompetent, with only two individuals having a history of arterial hypertension and one diagnosed with diabetes during hospitalization. Additionally, asthma was reported in two adults and two children. These findings were further supported by the Charlson Comorbidity Index (CCI) calculation, which indicated that most patients were otherwise healthy, lacking significant comorbidities. Despite this, frequent hospitalizations, oxygen therapy, and, in the most severe cases, mechanical ventilation were required, an occurrence that has never before been described in Split-Dalmatia County. Severe cases occurred sporadically, in contrast to the more consistent patterns observed in previously described infections among larger groups [8]. No clear link was found among the cases, and the factors that may have influenced this remain unproven. However, it could be assumed that the epidemic patterns of adenovirus and seasonal respiratory viruses have shifted due to the non-pharmaceutical interventions implemented in response to COVID-19 [17]. Also, a combination of factors can contribute to outbreaks like this, including genetic interactions and ecological and biological factors [18]. Complications such as pulmonary embolism and the need for mechanical ventilation occurred more frequently in previously healthy young male patients, a phenomenon that had not been observed before in this region.

In our study, the most common initial symptoms were fever (100%), sore throat (53.5%), diarrhea (48.6%), and vomiting (33.5%). Conjunctivitis was infrequently encountered. Those symptoms were more commonly identified in children compared to adults [32]. The laboratory results indicated a significant number of patients with elevated C-reactive protein (CRP) levels, with the highest value of 323 mg/L. The hospitalized patients had higher CRP levels and more complications than the non-hospitalized patients [33]. In recent years, reports have been published where severe hepatitis or acute liver failure was associated with HAdV in immunocompetent children [34]. In our study, elevated liver enzymes were observed in 12 adults and 3 children, but there were no cases of isolated severe hepatitis among children or adults. Elevated creatinine kinase and D-dimer were also observed, mostly in adults. The results of these laboratory parameters are related to a higher incidence of pulmonary embolism in young adults.

The predominant CT presentation was unilateral lobar pneumonia with multiple patchy ground glass opacities (23.5%) or lobar pneumonia with mixed opacities (17.6%). Bilateral lobar pneumonia was found in 1 (5.9%) patient, while, in a substantial portion of patients (11.8%), patchy consolidations or zones of ground glass opacification were detected. Similar CT findings were already described in the literature, as adenovirus pneumonia was confirmed to show bilateral multifocal GGO with patchy consolidations in the CT images. Adenovirus appears as multifocal consolidation or ground glass opacity (GGO), and GGO was more frequently observed in patients with adenovirus pneumonia than in those with other viral infections or bacterial infections [35]. In a study by Zhang et al., the predominant radiological finding of HAdV pneumonia was consolidation. Multilobular involvement, higher CT scores, and pleural effusion were found in more severe patients. The temporal changes in the radiological scores were consistent with the clinical findings [36]. A study by Chong on a small number of adult HAdV pneumonia patients reported bilateral patchy parenchymal opacities on chest radiographs and bilateral ground glass opacities with a random distribution, with or without consolidation, on HRCT images. These findings, however, are not specific to adenovirus pneumonia [37]. Severe adenovirus pneumonia in immunocompetent adults mainly appears as focal consolidation followed by rapid progression to bilateral consolidation, usually accompanied by adjacent GGO and pleural effusion, which may resemble bacterial pneumonia [38].

The CT presentation in our study was similar to the radiological presentation of COVID-19 pneumonia. Adenovirus and COVID cause both viral pneumonia, which might have similar and nonspecific imaging features. Both infections are commonly presented as multifocal patchy opacities at CXR or multifocal consolidations/ground glass opacities on chest CT. Therefore, the Brixia score and CT involvement score, the scoring systems developed and mostly used in COVID-19 pneumonia research, were chosen to quantify the extent of HAdV lung changes in our study.

In our study, pulmonary embolism was detected in five adults aged 26 to 36 and a 17-year-old child who had adenovirus pneumonia. All patients were previously healthy males with no medical history, but all of them had a history of smoking, except for the 17-year-old patient. Five patients had lobar pneumonia; one of them had unilateral multiple patchy ground glass opacities, and the other one had unilateral lobar pneumonia with multiple patchy ground glass opacities found on the CT. Three patients needed oxygen therapy. HAdV pneumonia-associated thrombosis is extremely rare. A hypothesis was put that thrombotic events may be related to viral endothelial injury and procoagulant activity [39]. To our knowledge, there is no reported case linking smoking to an increased risk of thromboembolism in patients with adenovirus infection.

Patients diagnosed with adenovirus pneumonia and elevated levels of C-reactive protein were predominantly treated with penicillin in combination with a beta-lactamase inhibitor, third-generation cephalosporins, or quinolones upon hospital admission. Antibiotics were administered in 42 (80.8%) of adult cases and 61 (45.9%) of pediatric cases. In the Appenzeller study, it was noticed that adenovirus infection was associated with elevated CRP concentrations, indicating that HAdV infection triggers an immediate inflammatory host response resembling invasive bacterial infection [40]. Based on the laboratory results, such as high CRP and, in some cases, elevated procalcitonin levels, as well as the radiological findings, it was difficult to distinguish whether there was a secondary bacterial infection accompanying HAdV pneumonia that resulted in the frequent, possibly unjustified, use of antibiotics, especially in severe forms of the disease. In Buonsenso’s study from 2024, 46% of HAdV-positive children received antibiotics but only 1.4% had confirmed bacterial infection [41]. There is a study that indicates a host-protein score (BV score), which combines the expression levels of TNF-related apoptosis-induced ligand, interferon gamma-induced protein 10, and C-reactive protein, helping to differentiate bacterial from viral infection [42]. Additional research is needed to reduce the use of antibiotics in patients with PCR-confirmed adenoviral infection without bacterial co-infection.

Methylprednisolone was given to 26 patients with severe or critical HAdV pneumonia intravenously, and 6 children were given inhaled budesonide. The mentioned therapy is compatible with the previous study from 2006, which showed that the treatment of pulse methylprednisolone therapy in adenovirus pneumonia rapidly improved respiratory distress [43]. However, during the Split-Dalmatia HAdV outbreak, the benefit of corticosteroid therapy was not proven, and larger studies are needed to evaluate the optimal treatment for severe forms of adenovirus infection.

Ribavirin and cidofovir are currently the most studied drugs for the treatment of adenovirus infection but without definitive proof of effectiveness. In our study, antiviral treatment with oral ribavirin was given to 18 adult patients, but there was no difference in the length of hospitalization between them and patients without ribavirin therapy. This could be explained by the fact that ribavirin was usually included in the therapy of patients with severe or critical pneumonia. So far, cases have been reported of community-acquired severe HAdV pneumonia, which was successfully treated with early administration of oral ribavirin [15,44]. The results from several case reports or small non-randomized studies have shown that cidofovir was effective in curing severe adenovirus infection [15,45]. The use of cidofovir for the treatment of HAdV infection requires careful monitoring due to its potential nephrotoxicity impact. Further studies are needed to determine the potential role of cidofovir in treating severe adenovirus infections.

This study has several limitations. First of all, it was a retrospective, observational study with limited control over the sampling of the population, as well as the nature and quality of the predictor variables. Additionally, successful serotyping of HAdV-isolates was performed only for a limited number of patients presenting with pneumonia; therefore, we can only assume that it was the same serotype. Serotyping and genome sequencing were unsuccessful due to the absence of specific pretreatment steps, which affected the ability to definitively identify specific adenovirus serotypes and genetic material that could explain why the outbreak was so severe.

## 5. Conclusions

The first case of HAdV infection in Split-Dalmatia County, Croatia, was confirmed on 25 October 2022, and the number of confirmed cases peaked in March 2023. A total of 185 patients were confirmed to be infected with HAdV, and 54 of them had pneumonia with the uncommon presentation of severe cases, mostly in otherwise healthy, young adult men. Following the above, adenovirus infection should be considered in the differential diagnosis of immunocompetent patients presenting with pneumonia or ARDS.

Adenovirus type 7 was isolated in all but one sample from patients with pneumonia, leading us to the conclusion that HAdV type 7 was responsible for the epidemic outbreak in Split-Dalmatia County.

Similarities in the radiological presentation between COVID-19 and adenovirus pneumonia were observed, mainly in terms of the multifocal patchy opacities on CXR or multifocal consolidations/ground glass opacities on chest CT and CXR. It was also observed that patients with severe or critical adenovirus pneumonia were at increased risk of developing pulmonary embolism. Thromboprophylaxis should, therefore, be considered in those patients. Treatment with ribavirin, glucocorticoids, and antibiotics did not shorten the course of the disease.

Genomic variants and recombinant viruses have the potential to trigger global outbreaks and lead to severe disease in both immunocompetent and immunocompromised individuals. Understanding the genetic mechanisms of adenovirus and other seasonal respiratory viruses is crucial for improving public health responses and preparedness for any future outbreaks with possible severe clinical manifestations.

## Figures and Tables

**Table 1:** Human-adenovirus-positive patients’ demographics and clinical features.

Characteristics *n* (%)	Total (*n* = 185)	Adults (*n* = 52)	Children (*n* = 133)	*p*
**Sex**				
Men	128 (69.2)	41 (78.8)	87 (65.4)	0.075 ^a^
Women	57 (30.8)	11 (21.2)	46 (34.6)	
**Median** **age (IQR), years**	8 (3, 20)	31 (23.8, 36.5)	5 (2.25–9)	<0.001
**Clinical manifestations**				
Fever	185 (100)	52 (100)	133 (100)	1 ^c^
Acute tonsillitis	99 (53.5)	19 (36.5)	80 (60.2)	0.004 ^a^
Diarrhea	90 (48.6)	29 (55.7)	61 (45.9)	0.226 ^a^
Vomiting	62 (33.5)	19 (36.5)	43 (32.3)	0.586 ^a^
**Pneumonia**	54 (29.2)	35 (67.3)	19 (14.3)	<0.001 ^a^
Mild	33 (17.8)	19 (36.5)	14 (10.5)	<0.001 ^a^
Severe	15 (8.1)	14 (26.9)	1 (0.8)	<0.001 ^c^
Critical	6 (3.2)	2 (3.8)	4 (3)	0.674 ^c^
**Hospitalized patients**	55 (29.7)	29 (55.8)	26 (19.5)	<0.001 ^a^
**Complications**				
Yes	14 (7.6)	9 (17.3)	5 (3.8)	0.002 ^a^
No	171 (92.4)	43 (82.7)	128 (96.2)	
**Type** **of complications**				
Pulmonary cavitations	1 (1.1)	1 (1.9)	0 (0)	0.281 ^c^
Pleural effusion	13 (7)	8 (15.4)	5 (3.8)	0.005 ^a^
Hospital infection	2 (1.1)	2 (3.8)	0 (0)	0.078 ^c^
**Pulmonary embolism**	6 (3.2)	5 (9.6)	1 (0.8)	0.007 ^c^
**Encephalopathy**	3 (1.6)	3 (5.8)	0 (0)	0.021 ^c^
**Therapy**				
Antibiotics	103 (55.7)	42 (80.8)	61 (45.9)	<0.001 ^a^
Corticosteroids	38 (20.5)	26 (50)	12 (9)	<0.001 ^a^
Ribavirin	18 (9.7)	18 (34.6)	0 (0)	<0.001 ^c^
Anticoagulants	20 (10.8)	19 (36.5)	1 (0.8)	<0.001 ^c^
**CCI**				
Mild	180 (97.3)	48 (92.3)	132 (99.2)	0.023 ^c^
Moderate	5 (2.7)	4 (6.7)	1 (0.8)	
Severe	0 (0)	0 (0)	0 (0)	
**Outcome**				
Favorable	179 (96.8)	50 (96.2)	129 (97)	<0.001 ^c^
Less favorable	5 (2.7)	2 (3.8)	3 (2.3)	
Death	1 (0.5)	0 (0)	1 (0.7)	
**Length of hospital stay in days, Md (IQR)**	6 (4, 9.75)	7 (5, 10)	6 (4, 8)	0.194 ^b^

AH—arterial hypertension, DM—diabetes mellitus, COPD—chronic obstructive pulmonary disease, CCI—Charlson Comorbidity Index; ^a^—Chi-square test; ^b^—Mann–Whitney U-test; ^c^—Fisher’s exact test; *p* < 0.05 (statistically significant).

**Table 2:** Laboratory test results of human-adenovirus-positive patients.

	Total	Adults	Children	*p*
Variable, Md (IQR)				
White blood cell count (10^9^/L)	*n* = 159 8 (5.9, 11.8)	*n* = 49 6.4 (5.3, 8.7)	*n* = 110 8.85 (6.1, 12.3)	<0.001 ^b^
Neutrophils (10^9^/L)	*n* = 157 69.2 (57.4, 76.5)	*n* = 50 75.85 (51.3, 72.8)	*n* = 107 66.4 (51.3, 72.8)	<0.001 ^b^
Lymphocyte count (10^9^/L)	*n* = 150 21.2 (15.9, 76.5)	*n* = 49 16.3 (11.3,20.3)	*n* = 101 25.2 (18.7, 39.8)	<0.001 ^b^
Platelet count (10^9^/L)	*n* = 148 192 (138.8, 259)	*n* = 49 133 (115, 165)	*n* = 99 220 (176, 274.5)	<0.001 ^b^
C-reactive protein (mg/L)	*n* = 161 70.5 (23.1, 89.6)	*n* = 50 82.8 (56.6, 135.9)	*n* = 111 40.9 (16, 71.8)	<0.001 ^b^
Liver function tests, N (%) Normal >2× larger	*n* = 124 109 (87.9) 15 (12.1)	*n* = 48 36 (75) 12 (25)	*n* = 76 73 (96.1) 3 (3.9)	<0.001 ^c^
Creatinine (µmol/L)	*n* = 116 56.5 (34.5, 84)	*n* = 47 88 (68.5, 103)	*n* = 69 38 (28, 53)	<0.001 ^b^
Na (mmol/L)	*n* = 107 135 (132,137)	*n* = 46 134 (130.25, 135)	*n* = 61 136 (133, 137)	0.006 ^b^
Lactate dehydrogenase (U/L)	*n* = 110 269 (191.3, 388.3)	*n* = 47 257 (175, 473.5)	*n* = 63 274 (202.5, 332.5)	0.703 ^b^
Creatine kinase (U/L)	*n* = 57 126 (81, 937)	*n* = 31 321 (103, 3165)	*n* = 26 91 (57.5, 133.5)	0.006 ^b^
D-dimer (mcg/L)	*n* = 41 3.749 (0.9, 3)	*n* = 33 1.2 (0.8, 2.6)	*n* = 8 5.4 (2, 10.3)	0.011 ^b^

Values are shown as median (Md) and interquartile range (IQR); ^b^—Mann–Whitney U-test; ^c^—Fisher’s exact test; *p* < 0.05 (statistically significant).

**Table 3:** Chest X-ray findings and median (IQR) of Brixia score index and Brixia score category, divided into two groups (adults and children).

	Total	Adults	Children	*p*
**Patients with** **CXR, *n* (%)**	112	46 (41.1%)	66 (58.9%)	
**CXR findings, *n* (%)**				
Normal CXR	57 (50.9)	13 (28.3)	44 (66.7)	<0.001 ^a^
Unilateral patchy opacity	13 (11.6)	5 (10.8)	8 (12.1)	0.996 ^a^
Unilateral patchy opacity and reticulations	7 (6.3)	5 (10.9)	2 (3)	0.105 ^c^
Unilateral reticulations	13 (11.6)	9 (19.6)	4 (60.6)	0.042 ^c^
Bilateral patchy opacities	4 (3.6)	2 (4.3)	2 (3)	0.637 ^c^
Bilateral reticulations	2 (1.8)	0 (0)	2 (3)	0.523 ^c^
Unilateral lobar pneumonia	8 (7.1)	3 (6.5)	5 (75.8)	1 ^c^
Bilateral lobar pneumonia	1 (0.9)	1 (2.2)	0 (0)	0.384 ^c^
Bilateral lobar pneumonia and multiple patchy opacities	1 (0.9)	1 (2.2)	0 (0)	0.384 ^c^
Unilateral lobar pneumonia and multiple patchy opacities	6 (5.4)	4 (8.7)	2 (3)	0.201 ^c^
**Brixia score, Md (IQR)**	0 (0.5, 3)	2 (0, 5)	0 (0, 2)	<0.001 ^b^
**Brixia score category, N (%)**				
Normal	58 (51.8)	16 (34.8)	42 (63.6)	0.018 ^c^
Mild	44 (39.3)	23 (50)	21 (31.9)	
Moderate	7 (6.3)	5 (10.9)	2 (3)	
Severe	3 (2.7)	2 (4.3)	1 (1.5)	

^a^—Chi-square test; ^b^—Mann–Whitney U-test; ^c^—Fisher’s exact test; *p* < 0.05 (statistically significant).

**Table 4:** Correlation between Brixia score and other laboratory parameters.

Laboratory Parameter	Total		Adults		Children	
	**Spearman’s Correlation Coefficient**	***p***	**Spearman’s Correlation Coefficient**	***p***	**Spearman’s Correlation Coefficient**	***p***
CRP (mg/L)	0.339	<0.001	0.32	0.03	0.2	0.12
White blood cell count (10^9^/L)	−0.277	0.005	−0.353	0.017	−0.117	0.380
Neutrophils (10^9^/L)	0.056	0.575	−0.104	0.493	−0.126	0.351
Lymphocyte count (10^9^/L)	0.021	0.835	0.107	0.483	0.255	0.063
Platelet count (10^9^/L)	−0.311	0.002	−0.275	0.067	−0.118	0.398
Creatinine (µmol/L)	0.154	0.163	−0.027	0.865	−0.195	0.221
Na (mmol/L)	−0.217	0.056	−0.245	0.118	0.016	0.928
Lactate dehydrogenase (U/L)	0.515	<0.001	0.530	<0.001	0.553	<0.001
Creatine kinase (U/L)	0.448	0.002	0.277	0.162	0.386	0.113
D-dimer (mcg/L)	0.320	0.061	0.225	0.242	0.667	0.148

**Table 5:** Median (IQR) of Brixia score index and Brixia score category in all human-adenovirus-positive patients, divided into two groups (hospitalized and non-hospitalized).

Variable	Total	Non-Hospitalized	Hospitalized	*p*
**Brixia score, Md (IQR)**	0.5 (0, 3)	0 (0, 1)	3 (1.25, 6)	<0.001 ^b^
**Brixia score category, *n* (%)**				
Normal	58 (51.8)	48 (72.7)	10 (21.7)	<0.001 ^c^
Mild	44 (39.3)	18 (27.3)	26 (56.5)	
Moderate	7 (6.3)	0 (0)	7 (15.2)	
Severe	3 (2.7)	0 (0)	3 (6.5)	

^b^—Mann–Whitney U-test; ^c^—Fisher’s exact test; *p* < 0.05 (statistically significant).

**Table 6:** Multivariate logistic regression results of the risk factors of less favorable outcomes or death in all-cause adenovirus infection.

Variable	OR	Estimate	95% CI	*p*
Male	11.189	2.415	(−2.565, 7.395)	0.342
Age	0.918	−0.085	(−0.214, 0.044)	0.197
CRP (mg/L)	1.014	0.013	(−0.004, 0.031)	0.131
Brixia score	1.548	0.437	(0.118, 0.755)	0.007

**Table 7:** The difference in the length of hospital stay in patients treated and not treated with antibiotics, ribavirin, and corticosteroids.

	Antibiotics-Used (*n* = 50)	Antibiotics-Not Used (*n* = 5)	*p*
**Length of hospital stay in days, median (IQR)**	6.5 (5, 10.75)	4 (3, 4)	0.346
	**Ribavirin-used** (***n*** = 18)	**Ribavirin-not used** (***n*** = 37)	***p***
**Length of hospital stay in days, median (IQR)**	7 (5, 10.5)	6 (4, 9)	0.444
	**Corticosteroids-used** (***n*** = 34)	**Corticosteroids-not used** (***n*** = 21)	***p***
**Length of hospital stay in days, median (IQR)**	7.5 (5, 11.75)	4 (4, 6)	**0.012 ^b^**

^b^—Mann–Whitney U-test, *p* < 0.05 (statistically significant).

## Data Availability

No new data were created or analyzed in this study. Data sharing is not applicable to this article.

## Supplementary Materials

The following supporting information can be downloaded at: https://www.mdpi.com/article/10.3390/pathogens13121114/s1, Table S1. Human-adenovirus-positive patient’s chest CT findings; Table S2. Demographic and clinical features of all non-hospitalized and hospitalized human-adenovirus-positive patients; Table S3. Laboratory test results of non-hospitalized and hospitalized human-adenovirus-positive patients; Table S4. Demographic, clinical, and laboratory characteristics of patients with pulmonary embolism; Table S5. Radiologist characteristics of patients with pulmonary embolism.
