# Supplementary material for: Clinical and Radiological Features of an Adenovirus Type 7 Outbreak in Split-Dalmatia County, Croatia, 2022–2023
Source: Pathogens. 2024 Dec 17;13(12):1114. doi: 10.3390/pathogens13121114 (PMC11678703; doi:10.3390/pathogens13121114)
Supplement: Supplementary file 1 [file pathogens-13-01114-s001.zip › Supplemental Table S1.pdf]

Table S1. Human Adenovirus positive patient's chest CT findings

	Total	Adults	Children	P^c
**Patients with CT scan N (%)**	N= 17	N=12	N=5	
**Chest CT scan findings**				
Unilateral lobar pneumonia	4 (23.5)	2 (16.7)	2 (40)	0.603
Unilateral lobar pneumonia and multiple patchy ground glass opacities	2 (11.8)	1 (8.3)	1 (20)	0.515
Unilateral lobar pneumonia and multiple patchy mixed opacities (GGO and consolidation)	3 (17.6)	2 (16.7)	1 (20)	1
Unilateral multiple patchy GGO (with or without interlobular septal thickening)	3 (17.6)	3 (25)	0 (0)	0.515
Bilateral lobar pneumonia with multiple patchy consolidations	1 (5.9)	1 (8.3)	0 (0)	1
Bilateral multiple patchy mixed opacities (GGO and consolidation)	2 (11.8)	2 (16.7)	0 (0)	1
Bilateral multiple patchy consolidations	2 (11.8)	1 (8.3)	1 (20)	0.515
**CT involvement score, median (IQR)**	6 (5.5, 11)	6 (5, 12)	8 (7, 9)	0.786
**CT involvement score category, N (%)**				
Mild	9 (60)	8 (61.5)	1 (50)	
Moderate	3 (20)	2 (15.4)	1 (50)	0.657
Severe	3 (20)	3 (23.1)	0 (0)	

c- Fisher's exact test, $p<0.05$ (statistically significant)
