# Supplementary material for: Clinical and Radiological Features of an Adenovirus Type 7 Outbreak in Split-Dalmatia County, Croatia, 2022–2023
Source: Pathogens. 2024 Dec 17;13(12):1114. doi: 10.3390/pathogens13121114 (PMC11678703; doi:10.3390/pathogens13121114)
Supplement: Supplementary file 1 [file pathogens-13-01114-s001.zip › Supplemental Table S2.pdf]

Table S2. Demographic and clinical features of all non-hospitalized and hospitalized Human Adenovirus positive patients

**Variable** **N (%)**	**Non-hospitalized** **(N=130)**	**Hospitalized** **(N=55)**	***p***
**Sex**			
Men	85 (65.4)	43 (78.2)	
Women	45 (34.6)	12 (21.8)	0.085[a]
**Age (Md, IQR)**	6 (3, 14)	19 (5.5, 34.5)	**<0.001[b]**
**Comorbidities**			
AH	0 (0)	2 (3.6)	0.092[c]
DM	0 (0)	1 (1.8)	0.273[c]
Asthma/COPB	2 (1.5)	2 (3.6)	0.584[c]
Obesity	0 (0)	7 (12.7)	**<0.001[c]**
Smoking habits	5 (3.8)	20 (36.4)	**<0.001[a]**
**Clinical manifestations**			
Fever	130 (100)	55 (100)	1[c]
Acute tonsillitis	80 (61.5)	19 (34.5)	**<0.001[a]**
Diarrhea	61 (46.9)	29 (52.7)	0.470[a]
Vomiting	43 (33.1)	19 (34.5)	0.847[a]
Pneumonia	13 (10)	41 (74.5)	**<0.001[a]**
Mild	13 (10)	20 (18.2)	**<0.001[a]**
Severe	0 (0)	15 (27.3)	**<0.001[c]**
Very severe	0 (0)	6 (10.9)	**<0.001[c]**
**Complications**			
Yes	0 (0)	14 (25.5)	**<0.001[c]**
No	130 (100)	41 (74.5)	

**Pulmonary embolism**	0 (0)	1 (1.8)	0.273[c]
**Encephalopathy**	0 (0)	(1.8)	0.273[c]
**Therapy**			
Antibiotics	53 (0)	50 (90.1)	**<0.001[a]**
Corticosteroids	4 (3.1)	34 (61.8)	**<0.001[c]**
Ribavirin	0 (0)	18 (32.7)	**<0.001[c]**
Anticoagulants	0 (0)	20 (36.4)	**<0.001[c]**
**CCI**			
Mild	129 (99.2)	51 (92.7)	
Moderate	1 (0.8)	4 (7.3)	
Severe	0 (0)	0 (0)	**0.028[c]**
**Outcome**			
Favorable	130 (100)	49 (89.1)	
Less favorable	0 (0)	5 (9.1)	**<0.001[c]**
Death	0 (0)	1 (1.8)	

AH- arterial hypertension, DM-diabetes mellitus, COPD- chronic obstructive pulmonary disease, CCI-Charlson Comorbidity Index; a-Chi square test; b- Mann Whitney U-test; c- Fisher's exact test; $p<0.05$ (statistically significant)
