# Supplementary material for: Clinical and Radiological Features of an Adenovirus Type 7 Outbreak in Split-Dalmatia County, Croatia, 2022–2023
Source: Pathogens. 2024 Dec 17;13(12):1114. doi: 10.3390/pathogens13121114 (PMC11678703; doi:10.3390/pathogens13121114)
Supplement: Supplementary file 1 [file pathogens-13-01114-s001.zip › Supplemental Table S3.pdf]

Table S3. Laboratory test results of the non-hospitalized and hospitalized Human Adenovirus positive patients

	**Non- hospitalized (N=130)**	**Hospitalized (N=55)**	***p***
**Variable**			
White blood cell count (10^9/L)	N=104<br>8.8 (6.1, 12.1)	N=55<br>8.5 (4.7, 9.6)	**0.007**[b]
Neutrophils (10^9/L)	N=103<br>78.85 (56.6, 74.6)	N=54<br>74.85 (62.8, 79.4)	**0.012**[b]
Lymphocyte count (10^9/L)	N=98<br>22.5 (17.1, 33.5)	N=52<br>18.6 (14.1, 29.6)	0.071[b]
Platelet count (10^9 /L)	N=94<br>205 (159, 275.3)	N=54<br>151 (118.3, 217.5)	**<0.001**[b]
C-reactive protein (mg/L)	N=106<br>43.85 (17.7, 76.6)	N=55<br>74.8 (40.5,135.4)	**<0.001**[b]
Liver function tests	N=72	N=52	
Normal	70 (97.2)	39 (75)	**<0.001**[c]
>2x larger	2 (2.8)	13 (25)	
Creatinine (μmol/L)	N=63<br>53 (37, 74.5)	N=53<br>60 (30, 96)	0.268[b]
Na (mmol/L)	N=55<br>135 (133.5, 137)	N=52<br>134 (130, 136.3)	**0.018**[b]
Lactate dehydrogenase (U/L)	N=59<br>205 (173, 279.5)	N=51<br>355 (269.5, 617)	**<0.001**[b]
Creatine kinase (U/L)	N=23<br>86 (52.5, 117.5)	N=34<br>559 (99.5, 3690.5)	**<0.001**[b]
D-dimer (mcg/L)	N=4<br>0.85 (0.68, 1.3)	N=37<br>1.8 (0.9, 3.3)	0.135[b]

b- Mann Whitney U-test; c- Fisher's exact test; $p<0.05$ (statistically significant)
