# Supplementary material for: Clinical and Radiological Features of an Adenovirus Type 7 Outbreak in Split-Dalmatia County, Croatia, 2022–2023
Source: Pathogens. 2024 Dec 17;13(12):1114. doi: 10.3390/pathogens13121114 (PMC11678703; doi:10.3390/pathogens13121114)
Supplement: Supplementary file 1 [file pathogens-13-01114-s001.zip › Supplemental Table S4.pdf]

Table S4. Demographic, clinical, and laboratory characteristics of the patients with pulmonary embolism (N=6)

	Sex	Age	Hospital stay (in days)	Comorbidities (HA, DM, asthma)	Smoking	Diarrhea	Vomiting	Acute tonsillitis	Pneumonia	Pleural effusion	WBC count (10^9/L)	Neutrophils (10^9/L)	Lymphocyte count (10^9/L)	PLT count (10^9/L)	CRP (mg/L)	Liver function tests	Creatinine (µmol/L)	Na (mmol/L)	LDH (U/L)	CK (U/L)	D-dimer (mcg/L)
**Patient 1**	male	31	9	No	Yes	No	No	No	Mild	No	12.7	59.4	28	362	71.9	Normal	60	134	1385	64	4.2
**Patient 2**	male	29	17	No	Yes	Yes	Yes	Yes	Severe	No	6.3	81	15.9	96	78.9	Larger x2	121	122	570	7816	2.9
**Patient 3**	male	26	14	No	Yes	Yes	Yes	No	Severe	Yes	10	75	13.7	354	17.3	Normal	55	135	233	NA	36.1
**Patient 4**	male	35	9	No	Yes	No	Yes	No	Mild	Yes	6.4	78	10	88	311.2	Normal	115	130	833	1619	2.2
**Patient 5**	male	17	4	No	No	No	No	No	Mild	No	2.6	50	38	103	63.5	Larger x2	58	140	1591	19885	10.3
**Patient 6**	male	36	11	No	Yes	No	No	No	Severe	Yes	5	78.2	17.4	85	64.9	Normal	74	128	296	104	6.8
