# Supplementary material for: Clinical and Radiological Features of an Adenovirus Type 7 Outbreak in Split-Dalmatia County, Croatia, 2022–2023
Source: Pathogens. 2024 Dec 17;13(12):1114. doi: 10.3390/pathogens13121114 (PMC11678703; doi:10.3390/pathogens13121114)
Supplement: Supplementary file 1 [file pathogens-13-01114-s001.zip › Supplemental Table S5.pdf]

Table S5. Radiologist characteristics of the patients with pulmonary embolism (N=6)

	Brixia score	CXR findings	CT involvement score	CT findings
**Patient 1**	2	Unilateral patchy opacity and reticulations	6	Unilateral multiple patchy GGO
**Patient 2**	4	Unilateral lobar pneumonia	5	Unilateral lobar pneumonia
**Patient 3**	3	Unilateral lobar pneumonia	5	Unilateral lobar pneumonia
**Patient 4**	2	Unilateral lobar pneumonia	6	Unilateral lobar pneumonia
**Patient 5**	6	Unilateral lobar pneumonia	6	Unilateral lobar pneumonia
**Patient 6**	10	Unilateral lobar pneumonia and multiple patchy opacities	22	Unilateral lobar pneumonia and multiple patchy ground glass opacities
